# Histopathology of the Host Cornea Following Penetrating Keratoplasty

**DOI:** 10.7759/cureus.39060

**Published:** 2023-05-15

**Authors:** Radha Vasan, Gurunadh S Velamakanni, Reena Bharadwaj, Satish K, Madhuri L Karri

**Affiliations:** 1 Department of Ophthalmology (Retired), Armed Forces Medical College, Bengaluru, IND; 2 Department of Ophthalmology, GSL Medical College, Rajahmundry, IND; 3 Department of Pathology (Retired), Armed Forces Medical College, Pune, IND

**Keywords:** fuchs’ dystrophy, immunohistochemistry, transmission electron microscopy, histopathology, penetrating keratoplasty

## Abstract

Introduction: To study the ultra-structural changes in the diseased corneal cells by histopathology, electron microscopy, and immunohistochemistry using conventional antisera and monoclonal antibodies with the ultimate goal of justifying pre-treatment and post-treatment advice and, if necessary, modifying the post-operative treatment for improved graft survival.

Methods: Thirty cases registered for penetrating keratoplasty were worked up for routine systemic and ophthalmic criteria. A full-thickness diseased cornea was subjected to histopathology after suitable staining and fixation, including electron microscopic and immunohistochemical studies where possible.

Results: The ages ranged from four to 60 years. The majority (26%) were in the age group of 31-40 years. The most frequent causes of corneal pathology that underwent keratoplasty include post-traumatic corneal scarring (40%), followed by pseudophakic bullous keratopathy (16.7%). In almost all cases, the histopathology confirmed the existing clinical diagnosis. Histopathology helped to confirm one doubtful case of Fuchs’ dystrophy and to contradict one clinical diagnosis of pseudophakic bullous keratopathy, which turned out to be epithelization of the anterior chamber.

Conclusion: The results underline the significance of the histopathological study of these corneal conditions to increase the post-surgical survival of the corneal graft.

## Introduction

Among the multiple causes of blindness, corneal opacity accounts for 8.2% [1]. A deeper comprehension of the corneal pathology enables better management and efficient outcomes from keratoplasty. Except for blood transfusions, keratoplasty is the most frequent and effective transplant performed [2]. The prognostic classification for penetrating keratoplasty (PKP) highlights the fact that it would be worthwhile to study the recipient factors [3], directing a better understanding of the disease process. A prospective study was therefore conducted on these cases of corneal blindness to ascertain the common causes and evaluate the histopathological characteristics of the diseased cornea under light microscopy, transmission electron microscopy (TEM), and immunohistochemical procedures.

## Materials and methods

A hospital-based prospective study was conducted at GSL Medical College & General Hospital, Rajahmundry, India, a tertiary care teaching hospital, to find out the common causes of corneal blindness, evaluate the importance of various microscopic techniques in studying corneal pathology, and improve the post-surgical survival of the corneal graft. The study was conducted for a duration of 12 months (from November 2020 to October 2021). A total of 30 patients were included in the study based on inclusion and exclusion criteria. Before starting the study, approval from the Institutional Review Board (IRB) and Institutional Ethics Committee (IEC) with approval number 728A-EC/728A-11/2020 was obtained. Written informed consent from all study subjects was obtained before data collection.

All cases registered for keratoplasty were worked up, which included the detailed history, the general examination, and the ophthalmic examination. The eye examination conducted was comprised of best-corrected visual acuity, lid examination for motility, eyelash alignment, state of the conjunctiva, and its wettability (Schirmer’s test). Then, the regurgitation on pressure over the lacrimal sac area was tested. Lacrimal syringing was performed. The extent, depth, opacification pattern, and vascularization of the cornea were all investigated. In the same manner, the extent and state of the anterior chamber and iris adherence (if present) were examined. It was followed by Goldmann applanation tonometry for intraocular pressure evaluation in cases with no surface irregularity. For the other few cases with an irregular corneal surface, Tono-Pen readings were taken. B-Scan ultrasonography was done to determine the condition of the posterior segment. Similar testing was performed on the fellow eye.

On receipt of the donation of a cornea, the recipient was called up for keratoplasty. The above-mentioned examinations were repeated afresh, and deviation, if any, from the pre-recorded details was noted. Per-operatively, in the operation theater, a trephine was applied to the recipient's cornea and rotated by hand. The central 7.0 mm to 7.5 mm of the diseased cornea was included in the cut, and to the greatest extent possible, the diseased or opacified cornea was removed. The remaining incision was completed with corneal scissors. The viscoelastic was injected into the anterior chamber, and the corneal button was cut out with scissors. As the button was removed, it was placed in a bowl containing Ringer's lactate.

The removed corneal specimen under study was labeled and dispatched to the pathology lab in a sterile tube with a full clinical summary and a presumptive diagnosis. In the laboratory, corneal buttons were chopped into halves or thirds. To facilitate easy accessibility, the corneal button was cut in half in all our cases. Each half was placed in a metal cassette that had pores in it, and these were placed in a large bin containing formalin. The cut tissue was left there to soak in the formalin.

The cassette with the tissue was then taken and emptied into an automatic tissue processor. Each of these jars contains formalin, varying grades of alcohol (70%, 90%, absolute, etc.), xylene, and paraffin wax as tissue-processing materials. Tissue embedding was done in paraffin wax. Hematoxylin and eosin stains were routinely used. Masson’s trichrome was also used in cases suspected of thickening of Descemet’s membrane. The prepared corneal specimen was studied under a light microscope. The ophthalmologist and the pathologist saw the prepared corneal slides together. The slides were studied by both of them in great detail for clinical and histopathologic correlation.

A portion of the second corneal hemisphere was preserved in 2% glutaraldehyde in 20 ml of the solution for further examination under transmission electron microscopy (TEM). Whenever required, this preserved corneal tissue was removed and thoroughly rinsed in phosphate buffer. It was then cut into micrometer sizes and processed for embedding. For the immunohistochemical procedure, immune-peroxidase methods were used.

Poorly prepared tissue at the laboratory, where histopathological differentiation, corneal specimen preparation for electron microscopy, and delineation of immunohistochemical markers were not possible, were excluded.

## Results

A total of 30 patients were enrolled in this prospective study. After a provisional pre-operative clinical diagnosis, the diseased corneas, removed at keratoplasty and subjected to histopathological examination, revealed that most occurrences of corneal blindness were in the age group of 31-40 years. Of these patients, 23 (77%) were males, and seven were females (23%).

The common cause of corneal blindness, and hence the commonest reason to use PKP [4], was corneal scarring secondary to trauma. These varied from simple post-traumatic scarring to complicated burns with four-quadrant vascularization. There were three re-grafts in this study (Table 1).

**Table 1:** Clinical diagnosis of the cases subjected to keratoplasty

Serial no.	Clinical diagnosis	N	%
1	Corneal opacity in children	03	10
2	Corneal scarring (post-traumatic) (04 leucoma cornea + 08 adherent leucoma)	12	40
3	Chemical burns	02	6.67
4	Microbial keratitis	03	10
5	Congenital hereditary corneal dystrophy	03	10
6	Pseudophakic bullous keratopathy (PBK)	05	16.67
7	Aphakic bullous keratopathy	02	6.67
Total		30	
Regrafts: 03 (Corneal dystrophy 02 + PBK 01)			

**Figure 1:**
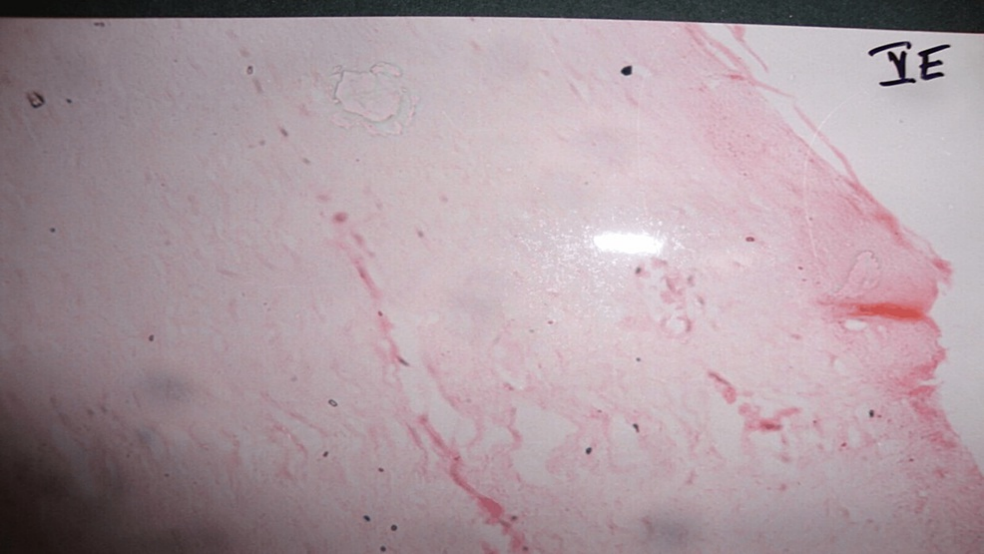
Histopathology of a corneal specimen: Fuchs’ dystrophy A rare case of Fuchs’ dystrophy was confirmed histologically by the thickened Descemet’s membrane with endothelial warts, which are diagnostic of the condition in contrast to post-cataract bullous keratopathy, wherein the same membrane is thinned out.

A presumptive clinical diagnosis of Fuchs’ dystrophy, for which a second diagnosis of pseudophakic bullous keratopathy was also kept in mind, surprisingly turned out to be Fuchs’ on histopathology (Figure 1).

**Figure 2:**
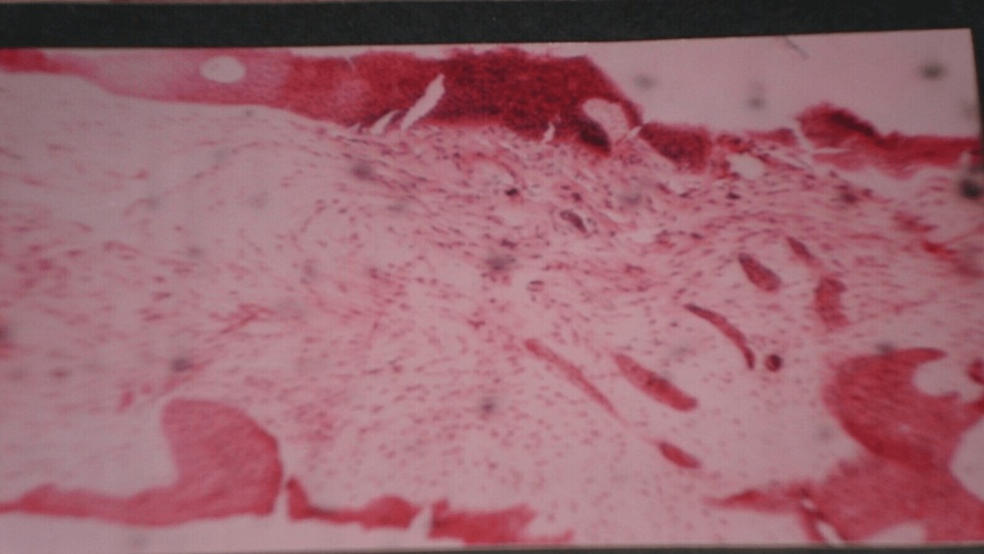
Histopathology slide of a corneal specimen showing anterior chamber epithelialization: epithelium up and down

Another case, where a diagnosis of pseudophakic bullous keratopathy was made, turned out to be epithelization of the anterior chamber (Figure 2).

Electron microscopy was in correlation with light microscopic findings in all cases. This result was all the more prominent in scarred corneas, such as adherent leucoma, where the corneal structure was disordered, the iris component was difficult to identify, and the endothelial cells showed elaborate polymegathism.

The corneal endothelial cells were positive for immunohistochemical stains, specifically vimentin and fibronectin, in all scarred corneas, such as in post-traumatic scarring and bullous keratopathy. Epithelial membrane antigen was found to be positive in chronic microbial keratitis but not in post-traumatic scarred corneas. Thus, the immunohistochemical analysis indicated that although vimentin was not detected in the normal cornea, post-wounding, corneal cells express vimentin; specifically, two days after wounding, epithelial cells exhibit vimentin. Although cytokeratins are also normally associated with vimentin, in the specimens in this study, cytokeratins were not isolated.

Histopathology confirmed the diagnosis in almost all cases, thus proving to be a useful tool for verifying the pre-operative clinical diagnosis and also arriving at the correct diagnosis, which helped to modify the post-operative treatment of the corneal graft recipients. Immunohistochemical analyses of corneal dystrophies were all the more revealing in the exactness of their diagnostic findings, specifically in granular corneal dystrophy and lattice dystrophy. Immunohistochemical analysis of macular corneal dystrophy did not yield any results, and the diagnosis was mainly made by history. It is also obvious that the prognostic grading for graft clarity, based on the indications for PKP, is useful to predict the outcome of the graft and indicates that in the category of poor prognosis, post-operative treatment modification is required.

## Discussion

Histopathology of the diseased corneal specimen, removed after keratoplasty, and the final diagnosis of the pre-existing corneal condition of the patient receiving a fresh corneal graft are the absolute goals of this study. An ultrastructural study gives an improved understanding of the disease process. The change the avascular, transparent cornea undergoes to become an opaque, vascularized structure is better understood by histopathology [5]. The result of any corneal disease is a corneal opacity, and all corneal opacities look alike [6]. Histopathology is not only a confirmatory tool in our final diagnosis, but it may also spring surprises.

Transmission electron microscopy (TEM) is a means of increasing the resolving power beyond that obtained by the ordinary light microscope. The most effective clinical use of TEM is encountered in selected situations when a light microscope cannot fully characterize a given lesion. TEM is a much less used diagnostic tool in ocular pathology and has specific diagnostic applications in tumor diagnosis. TEM is also a more rapid and less expensive means to diagnose ocular viral infections than viral culture. In this study, TEM added confirmatory evidence to the light microscopic findings [7].

The basic premise of an immunohistochemical procedure is to localize an antigen of interest in its cell of origin and, in most cases, to view the results at the light microscopic level. The diagnostic usefulness of this is that lesions derived from or showing differentiation toward a particular cell type retain an antigen that is useful in determining the lineage of that lesion. Consequently, the range of antibodies chosen for use in the lab is aimed at those that will help in distinguishing the derivation of poorly differentiated lesions [8]. The use of immunohistochemistry is more specific in cases of corneal dystrophies as compared to TEM [9]. As in the case of TEM, immunohistochemistry is also much more commonly used in tumor diagnosis and is very infrequently used in ocular pathology. However, immunohistochemistry did give valuable insight into the confirmation of post-traumatic corneal opacities. In granular corneal dystrophy, histochemical techniques positively identified the granular deposits in the stroma as abnormal proteins.

Clinically, the differential diagnosis of bullous keratopathy and Fuchs’ dystrophy is difficult to make in the advanced stage. If there is a history of cataract surgery, the provisional diagnosis considered is usually aphakic or pseudophakic bullous keratopathy as a complication of cataract surgery. Since Fuchs’ dystrophy is a rare entity in this region of the world [10], this entity is rarely entertained. But Fuchs’ is the most common corneal dystrophy to require keratoplasty and accounts for approximately 10% of all penetrating keratoplasties in the USA and Europe [11]. Histologically, both entities do not have much in common. Post-surgical bullous keratopathy showed thinning or absence of endothelium with bullae all over the stroma [12]. In Fuchs’, however, there is a thickening of the Descemet’s with endothelial warts, which are diagnostic of the condition [11]. In the present study, there was a case of Fuchs’ disease that showed characteristic endothelial warts. This patient was a woman and also had glaucoma in association, though it might be argued that the outcome could have been the same following the first penetrating keratoplasty. This case would not have been diagnosed but for the histopathology.

One of the specimens diagnosed as having pseudophakic bullous keratopathy on histopathology was found to have epithelization of the anterior chamber, which was unexpected. This was a case of penetrating corneal injury, which subsequently underwent cataract surgery, also subjecting this cornea to two insults that are associated with anterior chamber epithelization. Two dystrophies were diagnosed clinically. One was lattice dystrophy because of the characteristic findings in the other eye, and the other was macular stromal dystrophy, mainly because of history. This was confirmed by the histopathological study of the specimens as well.

The microbial keratitis was diagnosed mainly by the presence of inflammatory cells in the histopathological examination and vascularization [6], whereas, in the absence of the inflammatory cells, the specimen was diagnosed as due to non-infective keratitis in one of the children. The case of chemical burns was consistent with that in the literature [13]. Despite the energetic post-op treatment, after receiving confirmation from histopathology, the graft did not survive, and the patient has been put on long-term steroids, both topically and systemically, pending a fresh corneal transplant.

In two instances, the post-op regimen was guided by the histopathological report: firstly, in anterior chamber epithelization, and secondly, in cases of microbial keratitis, long-term steroids were instituted as the inflammatory cells were seen.

The main limitation was the smaller sample size. Further multicentric studies with a larger sample size and advanced research methods are required in the future to confirm the effectiveness and reliability of the present study.

## Conclusions

A post-operative histopathological examination of the recipient cornea may guide the treatment to be followed post-operatively to lower the possibility of graft rejection. The typical reasons for corneal blindness in this study were corneal scarring secondary to trauma. These varied from simple post-traumatic scarring to complicated burns with four-quadrant vascularization. Histopathology confirmed the diagnosis in almost all cases that had been given a presumptive clinical diagnosis before surgery. The surprises were: first, the case of Fuchs’ dystrophy, which, though presumptively diagnosed pre-operatively, despite its rarity, was upheld by histopathology; and second, the case of pseudophakic bullous keratopathy, which turned out to be anterior chamber epithelization. Thanks to histopathology, post-operative management could be modified to hasten healing, improve graft survival, and ultimately improve vision and quality of life.
